# 非小细胞肺癌中*ROS1*基因重排及其临床意义

**DOI:** 10.3779/j.issn.1009-3419.2013.12.09

**Published:** 2013-12-20

**Authors:** 陆亭 徐, 瑞景 赵, 增军 董, 铁年 朱

**Affiliations:** 1 200120 上海，赛信通（上海）生物试剂有限公司 Cell Signaling Technology China Branch, Shanghai 200120, China; 2 050017 石家庄，河北医科大学免疫学教研室 Department of Immunology, Hebei Medical University, Shijiazhuang 050017, China; 3 050082 石家庄，白求恩国际和平医院肿瘤科 Department of Medical Oncology, Bethune International Peace Hospital, Shijiazhuang 050082, China

**Keywords:** ROS1受体酪氨酸激酶, 间变性淋巴瘤激酶, 肺肿瘤, 克唑替尼, 个体化治疗, ROS1 receptor tyrosine kinase, Anaplastic lymphoma kinase, Lung neoplasms, Crizotinib, Personalized medicine

## Abstract

最近研究显示，包括非小细胞肺癌（non-small cell lung cancer, NSCLC）在内的许多恶性肿瘤存在ROS1受体酪氨酸激酶基因重排。*ROS1*基因重排作为一种新发现的NSCLC亚型，其发生率约占NSCLC的1%-2%，优势人群通常为年轻、不吸烟的肺腺癌患者，这些临床特征与ALK重排的NSCLC患者类似。体外实验和早期临床试验均显示，克唑替尼对ROS1重排阳性的癌症患者具有明显的抗肿瘤活性，治疗第8周和第16周时疾病控制率分别为76%和60%，治疗患者的总缓解率为56%。进一步了解*ROS1*基因重排在NSCLC发病中的作用，提高*ROS1*基因重排的检测技术，发现ROS1重排患者及研发特异性ROS1激酶抑制剂将为肿瘤个体化治疗增添新的篇章。

## Abstract (en)

Chromosomal rearrangements involving the ROS1 receptor tyrosine kinase gene have recently been described in multiple malignancies, including non-small cell lung cancer (NSCLC). ROS1 rearrangement defines a new molecular subset of NSCLC with the prevalence of ROS1 rearrangements around 1%-2%. ROS1-positive NSCLCs arise in young never-smokers with adenocarcinoma that are similar to those observed in patients with ALK-rearranged NSCLC. Crizotinib demonstrates *in vitro* activity and early clinical trial shows marked antitumor activity in ROS1-rearranged patients. The overall response rate is around 56% and the disease control rate at 8 weeks is about 76%. Further understanding the ROS1 fusions in the pathogenesis of NSCLC, methods to detect ROS1 rearrangements, and targeting ROS1-rearranged NSCLC patients with specific kinase inhibitors would lead to an era of personalized medicine.

在过去的十年里，针对肿瘤重要分子标志及信号传导途径治疗癌症的靶向药物研究取得了重大进展。根据分子标志筛选特定的疾病人群，应用阻断此标志的小分子化合物或单克隆抗体为肿瘤靶向治疗提供了个体化治疗模式的新思路，即发现新靶标，开发靶向治疗新药物，筛选靶向药物治疗患者。作为肺癌驱动基因之一的表皮生长因子受体（epidermal growth factor receptor, EGFR）酪氨酸激酶突变体的发现开启了非小细胞肺癌（non-small cell lung cancer, NSCLC）靶向治疗之门^[1]^。对EGFR酪氨酸激酶抑制剂（tyrosine kinase inhibitors, TKIs）的临床研究历程，从非选择患者到选择特定患者治疗群，从临床病理选择到分子标志物检测*EGFR*基因突变，而以EGFR-TKIs一线治疗*EGFR*基因突变患者的成功，标志性的奠定和推动了肺癌个体化治疗的进程^[2, 3]^。近两年针对间变性淋巴瘤激酶（anaplastic lymphoma kinase, *ALK*）融合基因的NSCLC靶向治疗的再次成功为肺癌驱动基因的研究锦上添花^[4-6]^，而越来越多的肺癌相关驱动基因的发现（如*ROS1*、*RET*、*KRAS*、*HER2*、*BRAF*、*PI3KCA*、*MEK1/2*、*MET*等），终将为NSCLC个体化治疗铺就蓝图^[7]^。本文对新发现*ROS1*融合基因靶点及针对这些靶点的检测方法以及药物治疗进展做一综述。

## 1 ROS1结构和生物学特性

受体酪氨酸激酶（receptor tyrosine kinase, RTK）是多种生长因子、细胞因子和激素的高亲和力细胞表面受体和胞内受体偶联的酪氨酸蛋白激酶总和。EGFR属于Ⅰ类RTK的EGFR/ErbB家族，而ALK属于X类RTK的LTK家族，ROS 1则属于Ⅱ类RTK的胰岛素受体家族。作为一个独特的受体酪氨酸激酶，ROS1在进化上相对保守。1982年，ROS1在UR2鸟肉瘤病毒中被确定为具有独特致癌作用的病毒原癌基因，此v-ROS1与*gag*基因发生融合而高表达具有致癌作用的蛋白质酪氨酸激酶融合蛋白p68gag-ROS^[8, 9]^。哺乳动物原癌基因*c-ROS1*位于第6号染色体q21区，全长cDNA包含44个外显子，编码2347个氨基酸，分子量为259 kDa。基本结构由胞外N-末端配体结合区（氨基酸1-1861）、跨膜区（氨基酸1862-1882）及胞内C-末端464个氨基酸构成的酪氨酸激酶活性区（氨基酸1883-2347）组成^[10]^。尽管ROS1为少数孤儿受体RTK，而缺乏对其配体的认知，但在蛋白质序列与结构分析上属于Ⅱ类RTK胰岛素受体家族。最近的氨基酸序列分析显示，在酪氨酸激酶区ROS1与ALK有49%的同源性，因为ALK酪氨酸激酶小分子抑制剂克唑替尼（crizotinib）的作用靶点在ALK激酶催化区的ATP结合位点，ROS1激酶催化区的ATP结合位点与ALK激酶催化区的ATP结合位点二者同源性高达77%^[11]^，因此ALK酪氨酸激酶小分子抑制剂克唑替尼（crizotinib）在治疗ROS1发生融合变异的NSCLC中具有明显疗效^[12-15]^。

尽管ROS1受体酪氨酸激酶在人体正常组织的功能尚未明了，但ROS1异位表达以及ROS1激酶的变异活化见于诸多肿瘤，如多形性神经胶质母细胞瘤、非小细胞肺癌以及肝外胆管癌，显示ROS1激酶活化诱导细胞的异常增殖与存活。在致病机理方面，尽管缺乏ROS1激活的合适配体或小分子激活剂，先前应用点突变和EGFR胞外受体部分构建的EGFR-ROS1嵌合蛋白表达技术业已证明，ROS1受体酪氨酸激酶参与激活多条下游信号转导通路，包括RAS-MAPK/ERK、PI3K/AKT/mTOR、JAK/STAT3以及PLCγ/IP3和SHP2/VAV3途径。前三者与肿瘤细胞增殖与存活有关，而后两者介导细胞形态转化和参与肿瘤细胞转移和迁移^[11, 16, 17]^。最近Gu等^[18]^在鼠白血病细胞系Ba/F3中高表达不同亚型的FIG-ROS1[fusion in glioblastoma (FIG)-ROS1]融合基因再次证明上述5种信号转导通路参与ROS1融合蛋白的致瘤作用。此外，ROS1激酶结合细胞骨架蛋白和细胞-细胞相互作用蛋白，直接或间接介导细胞骨架蛋白磷酸化，参与正常细胞的转化过程^[19]^。尽管类似其它RTK二聚化进而诱导其激酶活性异常活化的模式在报道中只是推测，但是多项试验^[20, 21]^证明活化诱导ROS1-RTK胞内激酶催化区酪氨酸自身磷酸化（Y2274和Y2334）在正常细胞转化至瘤作用中尤为重要。

## 2 *ROS1*融合基因

迄今为止，在非小细胞肺癌中已发现至少有9种不同的*ROS1*融合基因（图 1），包括最早发现于神经胶质母细胞瘤中的*FIG-ROS1*以及*CD74-ROS1*、*SLC34A2-ROS1*、*TPM3-ROS1*、*SDC4-ROS1*、*EZR-ROS1*、*LRIG3-ROS1*、*KDELR2-ROS1*和*CCDC6-ROS1*^[21-27]^。1987年在多形性神经胶质母细胞瘤细胞株U118MG中首先发现了*FIG-ROS1*融合基因^[28]^。*FIG-ROS1*融合基因的形成源于胶质母细胞瘤第6号染色体内*FIG*和*ROS1*基因间一段240 kb DNA片段缺失（6q21）导致*FIG-ROS1*基因融合并持续表达活化的ROS1 RTK激酶^[22]^。最近，美国Cell Signaling Technology公司的科学家应用自制的高敏感高特异兔单克隆抗体建立的免疫组织化学染色技术结合已知融合伙伴序列的RT-PCR技术在NSCLC中国人群中筛查*ROS1*融合基因时，除了检测到*SLC34A2-ROS1*和*CD74-ROS1*两种常见的融合基因外，还首次在NSCLC中发现了*FIG-ROS1*融合基因^[25]^。

**1:**
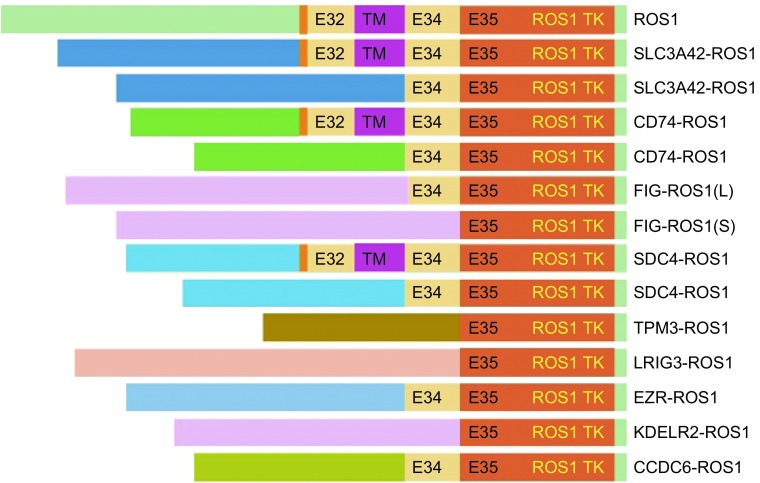
非小细胞肺癌中*ROS1*基因重排类型。外显子E32、E34和E35为ROS1融合基因断裂部位。TM为*ROS1*跨膜部分。 Type of *ROS1* rearrangement in non-small cell lung cancer. Exons 32, 34 and 35 are the *ROS1* fusion gene splicing domain. TM is transmembrane domain of ROS1.

早在2007年科学家们在应用酪氨酸激酶蛋白组学技术筛选肿瘤致癌基因时就发现了在NSCLC中存在*ROS1*基因重排现象^[5, 23]^。文中作者对41例NSCLC细胞株和150例中NSCLC患者的肿瘤样本进行了大规模的酪氨酸激酶筛选。结果除了发现*ALK*基因重排外，还发现1例NSCLC细胞株（HCC78）和1例患者的肿瘤样本中有*ROS1*基因重排；通过RT-PCR和DNA测序确定了两个独立的*ROS1*融合产物，即*SLC34A2-ROS1*和*CD74-ROS1*融合基因。在HCC78细胞系中，钠磷酸盐转运蛋白家族成员A2（SLC34A2）N-末端部分序列与ROS1部分跨膜区及全部胞内C-末端形成两种不同长度的SLC34A2-ROS1融合蛋白。尽管SLC34A2蛋白在人体组织中广泛表达，但SLC34A2在小鼠Ⅱ型肺泡上皮的表达参与了肺泡表面活性剂的合成，而*SLC34A2*基因突变与肺泡微小结石症形成有关，从而推断*SLC34A2-ROS1*基因重排可能参与了NSCLC的发病机制^[29]^。此HCC78细胞系后来被广泛应用于*ROS1*融合基因的研究。在1例NSCLC患者样本中，CD74的外显子1-6与ROS1部分胞膜和/或胞内C-末端氨基酸序列（包括外显子32/34到44）也形成两种不同长度的CD74-ROS1融合蛋白（图 1）。*CD74-ROS1*基因重排在*ROS1*基因重排中最为常见，约占30%。CD74具有巨噬细胞移动抑制因子（macrophage migration inhibition factor, MIF）受体功能，MIF-CD74相互作用参与非受体酪氨酸激酶介导的MAPK和Rho GTP酶活化，因而对巨噬细胞在宿主防御功能有调节作用^[30, 31]^。CD74同时作为一个MHC Ⅱ类分子相关蛋白，部分参与了MHC Ⅱ类蛋白的形成和运输。随后的体外和小动物体内转化实验证明SCL34A2-ROS1和CD74-ROS1具有致癌作用^[18, 32]^，而且后者还通过E-Syt1介导癌细胞的浸润和转移^[32]^。此后近两年如火如荼寻找肺癌驱动基因的研究^[14, 24, 26, 33]^进一步发现了*TPM3-ROS1*、*SDC4-ROS1*、*LRIG-ROS1*、*EZR-ROS1*和*KDELR2-ROS1*融合基因，并且均被证明在实验动物体内有致癌作用。

由于存在*SCL34A2-ROS1*基因重排的HCC78细胞系与上述其它*ROS1*融合基因在人或鼠真核细胞转染体系相比对克唑替尼只有中度敏感^[12-14]^，因此认为ROS1融合伙伴对ROS1酪氨酸激酶活性的影响因其亚细胞定位的差异可能反映出*ROS1*基因重组阳性肿瘤的临床病理表现和自然形成能力的潜在差异。比如，FIG-ROS1（S）和FIG-ROS1（L）之间的激酶活性和转化作用的差异归因于其在亚细胞的定位差异^[18]^。

与*EML4-ALK*基因重排产生的EML4-ALK融合蛋白定位于细胞质不同，不同的ROS1融合蛋白在细胞中的定位包括弥漫性胞浆分布、核周聚集、胞内微泡以及跨膜分布。SCL34A2-ROS1和CD74-ROS1为跨膜蛋白，而FIG-ROS1定位于细胞质或高尔基体。但是这些融合伙伴对NSCLC的致病作用还有待今后证明^[11, 18, 23]^。最后，值得注意的是上述不同类型的*ROS1*基因重排而非ROS1自身突变参与了NSCLC的致病作用，因为在NSCLC中没有发现任何ROS1激酶结构域有突变产生。相反，在结肠癌、卵巢癌、乳腺癌以及NSCLC非腺癌亚型中出现低频率的*ROS1*基因突变，但这些突变的意义尚且未知^[11, 23]^。

## 3 *ROS1*融合基因在NSCLC中的临床特征

*ROS1*基因重排与*ALK*基因重排在NSCLC中于2007年被同时发现^[23]^。由于ALK/MET多靶点RTK小分子抑制剂克唑替尼的成功研发，2008年全球首个*ALK*基因重排临床治疗试验在美国开始筛选和招募治疗NSCLC的*ALK*基因重排患者^[4, 34]^。尽管在NSCLC中只有3%-7% *ALK*基因重排，但临床治疗的巨大成功，使得ALK激酶抑制剂克唑替尼快速获得FDA批准用于*EGFR*无突变、ALK重排阳性的NSCLC的个体化治疗^[6]^。相反由于没有相应的ROS1激酶小分子抑制剂，针对*ROS1*基因重排的NSCLC临床治疗仅在最近相继报道^[12-15]^。这得益于体外实验证明克唑替尼能够有效抑制含有*SLC34A2-ROS1*基因重排而无任何ALK重排变异的NSCLC细胞系HCC78的生长及诱导其凋亡^[35]^。

*ROS1*基因重排代表一类新的独特的NSCLC分子亚型，其发生频率为1%-2%，远低于*EGFR*突变（10%-40%）和ALK重排变异（3%-7%），且存在一定的种族差异^[11, 36]^。但在EGFR/KRAS/ALK均阴性的人群中的发生率则可明显提高到5.7%^[36]^。*ROS1*基因重排阳性与ALK重排阳性的NSCLC患者具有相似性的临床特征，即为年轻、从不吸烟且为高恶性度趋势的肺腺癌患者^[12, 37]^。哈佛大学麻省总医院的Bergethon等^[12]^分析了18例ROS1阳性患者的临床资料。结果显示，NSCLC中ROS1阳性患者与ALK阳性患者的临床特征有着明显重叠性，ROS1阳性患者同样具有患病年龄年轻化（平均年龄49.8岁），从未吸烟的晚期（临床IV期）肺腺癌患者，而且亚洲患者占多数。此外，ROS1重排阳性的肿瘤多为低分化非典型性浸润癌。尽管麻省总医院在案治疗的浸润性NSCLC患者的总生存率（overall survival, OS）在ROS1重排阳性（663天）和阴性患者（607天）之间未见差别，但统计结果显示，ROS1重排阴性患者的预后较好。国内上海同济大学周彩存课题组^[38]^对8例ROS1重排阳性者进行了临床病例分析发现在中国人群中ROS1重排阴性的患者相比ROS1重排阳性预后好，但是在此小样本范围内两组患者间未能在年龄、性别及有无吸烟史上发现差别。

## 4 *ROS1*融合基因的检测

在各类肿瘤研究领域，基于分子标志物及癌症驱动基因的研究和临床试验取得了明显进展。*EGFR*基因突变、ALK基因融合变异、*ROS1*基因融合等分子靶点及其抑制剂的关系均进一步得到临床试验证实。然而，正如*ALK*基因重排只占肺癌的3%-7%，*ROS1*基因重排占肺癌比例更低1%-2%，因此，成功筛选病例是使用ROS1抑制剂进行个体化治疗成功的关键。目前，用于检测肺癌融合基因方法有多种，主要包括RT-PCR、荧光原位杂交和免疫组组织化学技术。

断裂荧光原位杂交（break-apart/Split FISH）技术是目前获得FDA批准的用于检测*ALK*融合基因的方法^[4]^，也是*ROS1*融合基因临床检测的常用方法^[12, 14, 24]^。FISH断裂探针为正常*ALK*或*ROS1*基因的5’-和3’-端不同标记有绿色和红色荧光的探针。正常情况下（无融合基因），这两个探针杂交结合于同一个基因，在荧光显微镜下显示为接近在一起的绿色和红色信号或二者重叠在一起的黄色信号。当存在基因重排时，绿色和红色信号因重组融合伙伴的置换而“断裂”显现为分离较远的单色信号。理论上，FISH适用于检测任何一种融合伙伴及任何类型基因重排。但是，如果染色体倒位或缺失较小，两个探针相距小于12M bp，显微镜下分裂信号微小，检测具有挑战性，因此，针对RT-PCR确诊或IHC阳性的融合基因而FISH检测结果却出现假阴性。FISH缺点包括相对高的成本，需要专用设备，试剂昂贵，较长的处理时间，以及高水准的专业诊断技术，因而不为大多数临床病理实验室所具备。另外，FISH方法不能鉴别融合基因的融合伙伴，是相对较低通量的检测。Bergethon等^[12]^应用断裂FISH技术，对1, 073例NSCLC患者进行筛查，发现18例*ROS1*基因重排病例，阳性率约为1.7%。进一步通过RT-PCR证实含有*SLC34A2-ROS1*和*CD74-ROS1*融合基因。Takeuchi等对1, 476例日本NSCLC患者使用FISH技术确定了13个*ROS1*融合基因，阳性率为0.9%（包括*CD74-ROS* 11例，*SLC34A2-ROS* 11例，*SDC4-ROS* 13例，*TPM3-ROS* 12例，*LRIG3-ROS* 11例和1例未知）。同样，Davies等^[14]^在最近的一项研究中应用FISH技术发现*ROS1*基因重排阳性率为1.2%（428例中有5例阳性）。

RT-PCR技术简单快速准确。染色体倒位或缺失融合致使该基因DNA序列具有特殊性，PCR引物只与融合基因转录子相结合，从而使得此方法具有高灵敏度，并可确定融合基因的融合伙伴。同时，该方法适用于微量组织标本，比如病人痰标本或用支气管镜取得的活检组织，因而RT-PCR技术筛选和确认肺癌基因重组具有独特的优势。RT-PCR缺点：需要足够的高质量没有污染的RNA，这在福尔马林固定和石蜡包埋的常规肿瘤标本通常很难获得。此外，大量潜在的ROS1融合蛋白及其变体的存在，则需要足够大的PCR引物组以涵盖所有潜在的*ROS1*重组基因。还有，RT-PCR结果常常出现假阳性。因此，该方法难以成为常规临床检测方法。上海同济大学周彩存课题组^[38]^应用多重RT-PCR技术筛选了392例中国人NSCLC患者的FFPE标本，发现8例*ROS1*基因重排，重排率约为2.0%。另一项研究^[39]^对202例中国人肺腺癌患者进行RT-PCR筛选，ROS1阳性率为1%。

免疫组织化学染色在临床病理实验室的应用极为普遍，而且免疫组化具有低成本、简便快速和不失准确性，对包括*ALK*、*ROS1*基因重排的患者亚群筛选是一个理想的工具。但IHC对临床疾病生物标记分子的检测需要高质量的单克隆抗体，尤其对组织中低水平蛋白表达的检测。比如，早期使用鼠ALK单克隆抗体来诊断NSCLC患者ALK融合蛋白的检测效果很不理想，原因是ALK融合蛋白在肺癌中的表达比在ALCL中要低很多。新近采用高敏感高特异的单克隆抗体所进行的IHC染色方法检测*ALK*、*ROS1*基因重排，结果发现与断裂FISH技术相比，准确度可达到100%，特异性高达99%^[25, 40]^。IHC缺点：IHC染色对不同组织测试点之间存在差别，并且IHC不能区分ROS1野生型和重排型。

总之，虽然FISH是目前肺癌融合基因诊断的金标准，但是每个方法都难免有优点和缺点（表 1）。我们应根据每种技术的敏感性、特异性及其检测局限性将它们应用到临床检测中。尽管*ROS1*基因重排阳性率很低，但如果以临床病理特征为基础来检测*ROS1*融合基因，在排除*EGFR*突变、*KRAS*突变、*HER*突变、*p53*突变以及ALK融合变异后，正如*EML4-ALK*融合基因的检出率由占全部NSCLC的3%上升到约占25%^[41]^，ROS1融合变异的检出率也会大大增加，从而为肺癌个体化治疗提供有效的诊断基础。

**1:** *ROS1*基因重排检测方法 Detection methods of *ROS1* rearrangement

Test	Advantage	Disadvantage
Split FISH	Applicable for any partner genes	Expensive
	Applicable to FFPE tissues	Difficult to interpret results
		Need for technical expertise
RT-PCR	Minimum of sample	Samples easy to be contaminated
	Identification of fusion partners	Difficult to obtain high-quality of RNA
		Difficult to discover new fusion partners
IHC	Cheap	Low expression of ROS1 fusion protein
	Routine pathological diagnosis assay	Need high-affinity antibody
	Applicable to FFPE tissues	Fail to identify fusion partners
FFPE: formalin-fixed paraffin-embedded.		

## 5 ROS1靶向治疗NSCLC

### 5.1 ROS1激酶抑制剂

ROS1受体酪氨酸激酶是一个重要的癌症驱动基因，所以靶向定位抑制它的激酶活性，可以提供更有效的和有选择性的治疗由它衍生出来的癌症。RTK选择性抑制剂的研发是当今靶向治疗NSCLC领域的一个热点。早期研发的Staurosporine等能高效抑制ROS1激酶活性（IC_50_=0.9 nM），但其选择性较差，因而限制了其临床应用^[42]^。AZD1480最近被证明在体外可抑制ROS1激酶活性，在动物体内可明显缩小ROS1融合所致肿瘤大小，结合其实质为JAK1/2特异性抑制剂，可阻断STAT3信号通路，从而具有抑制*ROS1*基因重排诱导的肿瘤发生^[43]^。再有，El-Deeb等^[44]^合成了针对ROS1激酶的小分子抑制剂并对其在45种以上不同的受体酪氨酸激酶的抑制作用进行了筛选，发现两个小分子抑制剂KIST301072和KIST301080具有高特异的抑制ROS1激酶活性（IC_50_分别为199 nM和209 nM），其临床前研究尚需进一步得到肯定（表 2）。

**2:** ROS1酪氨酸激酶剂的IC_50_ IC_50_ of selected ROS1 tyrosine inhibitors

Drug	Manufacture (ROS1)	IC_50_	IC_50_(ALK)	Reference
TAE684	Novartis	10 nM	< 10 nM	35
Crizotinib	Pfizer	1.7 nM	0.5 nM	45
AP26113	Ariad Pharm	1.9 nM	0.69 nM	46
WZ-5-126	Ambit Bio	8.2 nM	3.4 nM	35
ASP3026	Astellas	n/a	n/a	46
AZD1480	AstraZeneca	n/a	n/a	43
KIST301072	Korea Insitute	199 nM		44
KIST201080	of Sci. and Tech	209 nM		44

### 5.2 ALK激酶抑制剂

由于目前针对ROS1酶特异抑制剂的研究尚不成功，而氨基酸序列分析发现，ALK和ROS1激酶结构域间约有49%氨基酸序列同源性，而且ALK和ROS1激酶结构域ATP-结合位点区域的同源性更高达77%^[11]^。因此，ALK激酶抑制剂可能还抑制ROS1激酶活性。

#### 5.2.1 临床前研究

在发现NSCLC中存在*ALK*基因重排后不久，科学家即发现ALK激酶抑制剂对ROS1激酶有抑制作用。McDermott等^[35]^在对NVP-TAE684（一种特异ALK酪氨酸激酶抑制剂）处理的602个肿瘤细胞系进行筛查时发现，包括NSCLC、神经母细胞瘤和间变性大细胞淋巴瘤细胞系在内的10个肿瘤细胞系对TAE684治疗敏感，其中包括SLC34A2-ROS1重排阳性而无任何ALK重排的NSCLC细胞株HCC78^[23]^，这一结果提供了ALK激酶抑制剂对ROS1酪氨酸激酶有抑制作用的直接证据。随后的试验再次证明，TAE684还具有抑制FIG-ROS1融合变异转染的Ba/F3细胞系^[18]^。此外，ALK/MET多靶点激酶抑制剂克唑替尼也可适度或全部抑制上述HCC78细胞系或CD74-ROS1转染的HEK293细胞系^[12]^、SDC4-ROS1转染的Ba/F3细胞系^[14, 25]^以及EZR-ROS1转染的小鼠NIH3T3细胞系^[33]^。上述研究进一步证明ALK激酶抑制剂的作用机理是可逆性地结合于ROS1激酶结合区，从而抑制了Tyr2774的自身磷酸化，阻断了激酶活化诱导的下游信号活化通路^[11]^。

#### 5.2.2 临床研究

Bergethon等^[12]^最先成功报道ALK激酶抑制剂克唑替尼治疗ROS1重排变异单病例NSCLC患者。患者为31岁年轻男性，从不吸烟，属腺癌亚型，未见*EGFR*突变和ALK重排。对厄洛替尼治疗无效，且逐步恶化。经额外基因检测为ROS1重排阳性，并开始克唑替尼标准治疗（剂量为250 mg，每日两次）。不到1周，患者临床症状明显改善，2周后缺氧消失，8周时CT扫描肺部肿瘤病灶几乎完全消失。患者继续用药6个月无复发迹象。在此基础上，同一课题组在今年6月的芝加哥2013年ASCO年会上报道了克唑替尼治疗*ROS1*基因重排较大样本的Ⅰ期临床试验，包括31例ROS1阳性晚期NSCLC患者。在治疗8周和16周时患者的疾病控制率分别达到76%和60%。治疗总缓解率为56%，包括2例完全缓解，12例部分缓解和8例疾病稳定。6个月无进展生存率达到71%。这表明，克唑替尼对ROS1重排阳性NSCLC患者有明显治疗效果^[13]^。同样结果见于另外2例报道，在患者对EGFR-TKI一线治疗无效的基础上，筛查到ROS1重排变异，结果患者对克唑替尼治疗8周后出现奇效，其中1例患者证明存在有SDC4-*ROS1*基因重排^[14, 15]^。克唑替尼对ROS1重排阳性肿瘤患者的临床疗效尚需更大样本、更多临床研究单位参与和证实。随着新的ALK激酶抑制剂的出现^[45, 46]^，其对ROS1融合变异的NSCLC患者的临床Ⅰ期试验也正在进行中。

以上充分体现了实施快速分子诊断技术发现新的肺癌亚型，以制定针对特定病人的个体化治疗的重要性。但这项研究的成功也使得临床应用分子诊断越来越复杂，从常规检测的*EGFR*、*KRAS*突变，到ALK重排、再到ROS1重排，以及今后包括的新靶点的检测。目前临床试验使用的*ROS1*基因重排的各种检测方法尚未得到商业化。

## 6 展望

以分子分型为基础的肿瘤靶向治疗代表了肿瘤治疗的最新发展方向，而根据基因检测进行分子分型、进而合理选择治疗方案实施个体化疗必将成为今后肺癌临床治疗的主流。ROS1重排阳性作为一个新的NSCLC分子亚型，虽然罕见，但已经成为一个有效的治疗靶点，并期待今后临床试验结果给予更完善更全面的佐证。未来的工作将包括检测ROS1融合变异的最佳方法、完善ROS1重排阳性肿瘤的治疗群、鉴定ROS1融合蛋白下游信号通路、发现ROS1 RTK抑制剂出现抵抗的机理以及研发新的特异性ROS1酪氨酸激酶抑制剂。
